# Mental health at different stages of cancer survival: a natural language processing study of Reddit posts

**DOI:** 10.3389/fpsyg.2023.1150227

**Published:** 2023-06-23

**Authors:** Ioana R. Podina, Ana-Maria Bucur, Diana Todea, Liviu Fodor, Andreea Luca, Liviu P. Dinu, Rareș F. Boian

**Affiliations:** ^1^Laboratory of Cognitive Clinical Sciences, University of Bucharest, Bucharest, Romania; ^2^Department of Applied Psychology, University of Bucharest, Bucharest, Romania; ^3^Interdisciplinary School of Doctoral Studies, University of Bucharest, Bucharest, Romania; ^4^International Institute for The Advanced Studies of Psychotherapy and Applied Mental Health, Babeș-Bolyai University, Cluj-Napoca, Romania; ^5^Evidence Based Psychological Assessment and Interventions Doctoral School, Babeș-Bolyai University, Cluj-Napoca, Romania; ^6^Human Language Technology Research Center, University of Bucharest, Bucharest, Romania; ^7^Faculty of Mathematics and Computer Science, University of Bucharest, Bucharest, Romania; ^8^Department of Computer Science, Babeş-Bolyai University, Cluj-Napoca, Romania

**Keywords:** cancer survivors, social media, natural language processing, mental health, Reddit

## Abstract

**Introduction:**

The purpose of this study was to use text-based social media content analysis from cancer-specific subreddits to evaluate depression and anxiety-loaded content. Natural language processing, automatic, and lexicon-based methods were employed to perform sentiment analysis and identify depression and anxiety-loaded content.

**Methods:**

Data was collected from 187 Reddit users who had received a cancer diagnosis, were currently undergoing treatment, or had completed treatment. Participants were split according to survivorship status into short-term, transition, and long-term cancer survivors. A total of 72524 posts were analyzed across the three cancer survivor groups.

**Results:**

The results showed that short-term cancer survivors had significantly more depression-loaded posts and more anxiety-loaded words than long-term survivors, with no significant differences relative to the transition period. The topic analysis showed that long-term survivors, more than other stages of survivorship, have resources to share their experiences with suicidal ideation and mental health issues while providing support to their survivor community.

**Discussion:**

The results indicate that Reddit texts seem to be an indicator of when the stressor is active and mental health issues are triggered. This sets the stage for Reddit to become a platform for screening and first-hand intervention delivery. Special attention should be dedicated to short-term survivors.

## 1. Introduction

Most cancer survivors outlive their cancer for 10 years or longer (Haun et al., 2014). Hence, the concern for a good quality of life after diagnosis is not limited to physical health only. Both depression and anxiety are frequent complications of cancer, with one-third of cancer survivors being diagnosed with one or both conditions (Haun et al., 2014). This not only alters quality of life, but it even reduces cancer survival rates (Stark and House, 2000; Smith, 2015). It is unclear whether depression and anxiety symptoms are equally bad, less bad, or worse at different phases of survivorship. Research has found that depression and anxiety peak around the time of diagnosis or the early stages of cancer treatment and subside with time (e.g., Bergerot et al., 2017), but there are studies that contradict these results and indicate that symptoms do not subside in time (Breidenbach et al., 2022).

Monitoring patients is time and resource consuming and often can only be done when survivors come in for their annual visit. This explains why few studies have examined psychological distress beyond 5 years after diagnosis (Syrowatka et al., 2017; Yi and Syrjala, 2017; Carreira et al., 2018; Kuba et al., 2019). Fortunately, cancer survivors have developed a preference for using social media platforms to communicate their opinions, mental states, and everyday activities, resulting in user-generated material that is typically unavailable to healthcare providers.

One particular advantage of mining social media platforms for mental health content is the valuable insight obtained from a first-person stance (Guntuku et al., 2017). On this note, recent research has introduced the prospect of employing social media data for mental health screening and/or early detection (Bucur and Dinu, 2020; Bucur et al., 2021). This potential can be further harnessed by utilizing Natural Language Processing (NLP) tools to analyze, understand, and infer the meaning of user-generated texts on social media (Calvo et al., 2017; Guntuku et al., 2017).

By leveraging NLP models, which predominantly utilize binary classification, it becomes possible to categorize messages from platforms like Reddit or other social media networks, distinguishing between depressive and non-depressive content. To achieve accurate classification, a ground truth data set with annotated texts from clinical and non-clinical individuals is often required for binary classification. Subsequently, social media posts are classified against these datasets. Remarkably, Shen and Rudzicz (2017) were able to distinguish anxiety-laden messages on mental health subreddits from control subreddits with 98 percent accuracy. Similarly, NLP algorithms have been successful in detecting depression from Twitter with 70 percent accuracy (Choudhury et al., 2013) and bipolar disorder from Reddit with 86% accuracy (Sekulic et al., 2018).

In this study, we explored the capabilities of NLP to detect mental illness in a group of volunteer cancer survivors. We aimed to investigate whether social media content, in our case Reddit content, is an appropriate environment to determine which text is depression and anxiety-loaded and whether its frequency varies with cancer survivorship stage. We divided our sample into short-term (0–2 years after diagnosis), transitioning (> 2–4 years after diagnosis), and long-term survivors (≥ 5 years since diagnosis) based on past literature (Götze et al., 2014; Okado et al., 2016; Wang et al., 2016). The short-term survival phase includes adjustment to diagnosis, treatment, and treatment-related toxicity. The transition phase is about departing from patient status and a high risk of cancer recurrence to improved survival prospects and long-term survivor status. Long-term survivor status ultimately means a more complete resumption of normal life activities and a minimal likelihood of cancer recurrence.

Reddit was chosen as a platform because it allows anonymous profiles, enabling honest conversations (Choudhury and De, 2014; Ammari et al., 2019) that would be difficult to have on other social media sites (Newman et al., 2011). Despite the fact that Reddit posts are not accompanied by formal clinical diagnoses, Reddit has several advantages over traditional mental health datasets. The data are publicly available and allows for cross-temporal comparisons.

Our hypothesis was that in the short-term survival phase, there would be more Reddit content labeled as depressed or anxious than in the transitional and long-term survival phases. In this phase, the memory of the diagnosis, the side effects of treatment, and the fear of recurrence are still fresh and very much present (Naughton and Weaver, 2014). For these reasons, short-term survival is believed to be a vulnerable time for mental health (Yi and Syrjala, 2017). Similarly, we predicted that the long-term survival phase would have the least depressed and anxious content of the three as cancer recurrence probability decreases with each year after diagnosis (Mahvi et al., 2018).

These assumptions are also based on the diathesis stress model (Zuckerman, 1999), a psychological framework for understanding how stress affects our mental health. According to this model, everyone is susceptible to some degree to depression, anxiety, and other mental problems and illnesses. Excessive stress can cause symptoms that were not present before. Similarly, psychological problems triggered by stress usually subside once the stressor is eliminated or removed (Zuckerman, 1999; Segerstrom and O’Connor, 2012). Further theoretical background is based on the *cancer survivor adaptation model* (CSA; Naus et al., 2009), a theoretical framework that discusses adjustment to cancer survivorship as a life-long process that happens gradually.

This is the first study of its kind to employ NLP tools and analytics, such as automatic methods for depression detection and lexicon-based methods for identifying content portraying anxiety in cancer survivors. The depression and anxiety-loaded content was further explored via topic analysis to understand the subjects that are discussed on Reddit by cancer survivors. Additionally, we searched for texts that might use terms connected to cancer and suicide, as well as looked into the general sentiment of the discourse (positive vs. negative valence). In addition, we retrieved personal concerns related to death, school, job, money, the body, and sex drive. These categories were selected in order to gain a deeper understanding of the lives of cancer survivors, as ample research indicates that survivors are concerned about their mortality prospects (Soleimani et al., 2020), educational, financial, and occupational challenges during and after treatment (Sisk et al., 2020), as well as body, sex drive (Sacerdoti et al., 2010; Shankar et al., 2017) and side effects of cancer treatments.

We expect that quantifying text differences between short-term, transitional, and long-term survivors will provide valuable insight into the utility of social media content in complementing cancer survivors’ mental health screening and will inform the provision of responsive care to Reddit cancer survivor communities.

## 2. Methods

### 2.1. Participants and data collection

We collected data from 187 Reddit users who had been given a cancer diagnosis, were currently receiving treatment, or had finished their course of treatment. Participants were recruited through announcements on cancer-related subreddits (e.g., r/cancer, r/breastcancer), or through individual invites sent by the research team to users of the aforementioned subreddits. The participants were invited to respond to an online survey on their demographics, year of cancer diagnosis, and other aspects relevant to the disease and treatment (see the section “3”).

Despite there being 304 respondents who filled out the survey, only 187 consented to have their Reddit posts’ data extracted. The survey data were acquired using an online self-report questionnaire made available on the QuestionPro platform (Free Online Survey Software and Tools|QuestionPro®, n.d.). Using the Python Reddit API Wrapper (Boe, 2016), we collected the publicly available Reddit submissions of users who consented to data extraction. Both comments and posts of the consenting enrolled users were extracted from all the subreddits in which the users were active, with data collection not being limited only to cancer-related subreddits.

Having inquired about the year of diagnosis, we were able to split Reddit posts into short-term (0–2 years since diagnosis), transitional (> 2–4 years since diagnosis), and long-term survivorship intervals (≥ 5 years since diagnosis) as per Götze et al. (2014), Okado et al. (2016), and Wang et al. (2016). Given the fact that this study analyzes the data at the user level, all the texts from each user were concatenated, and users with less than 50 words were removed from the dataset in order to keep the users that have enough textual information for the analyses. This is a procedure similar to Crossley et al. (2016). The cut-off was chosen by taking into account the number of words and not the number of posts because on the Reddit platform a post can have up to 40,000 characters, and just one post can have sufficient information to be analyzed.

To identify the languages used in texts, we employed automatic open-source tools, specifically the polyglot package available for Python. The model estimated that approximately 95% of the posts were written in English. The manual analysis of the remaining 5% of the data revealed that less than 1.6% of the texts were written in languages other than English. Moreover, due to the large number of samples, a manual inspection of all the automatically labeled data was unsuitable. A manual inspection of a subset of the content labeled as depression-loaded was performed when choosing suitable names for the topics extracted by the Latent Dirichlet Allocation model.

Due to the nature of the data collection process, information was collected from multiple stages of survivorship (short-term, transition, and long-term) for some participants. In order to avoid violating the independence of observations assumption (i.e., having participants’ data from multiple stages of survivorship), users that had data from more than one period of survivorship, were kept in the period in which they had the most posts, and removed from other periods in which they had fewer posts. This filtering of users was performed while keeping a balanced number of users across stages of survivorship. We note that multilevel longitudinal analyses were not possible as only around 15% of the volunteers had posts in more than one cancer survival interval, and typically their posts extended to no more than two cancer survival intervals.

This study analyzes data from 77 users in the short-term phase, 55 users in the transition phase, and 55 users in the long-term survival phase. The average number of posts per participant was 388, with 24.376 posts from survivors in the short-term group, 16.898 posts from survivors in the transition group, and 31.250 posts from survivors in the long-term group. The analysis code is provided in the Supplementary material.

### 2.2. Ethics declarations

The project received approval from the Research Ethics Committee, and all volunteering participants provided written informed consent prior to inclusion in the study. All methods were performed in accordance with the relevant guidelines and regulations.

### 2.3. Reddit discourse analysis

Prior to text analysis, the posts and comments from users were pre-processed by converting them to lowercase and removing stop words and URLs from all the texts. The cues related to mental health from the cancer survivors’ texts were extracted using lexicon-based and automatic models. The following features were analyzed from the social media discourse of cancer survivors: depression-loaded content; frequency of anxiety-related words; frequency of suicide keywords; sentiment analysis (frequency of positive and negative emotion words); frequency of words related to personal concerns; and frequency of cancer-related terms. All the text features were computed at the user level by concatenating all the posts from each individual.

#### 2.3.1. Lexicon-based discourse analysis

Cancer-related terms were extracted from users’ Reddit posts using Cancer.org‘s glossary of 943 words, which is one of the resources used by Jung et al. (2021) to build a breast cancer lexicon. Similarly, the words related to suicide from Sawhney et al. (2018), such as *suicidal*, *want to die*, *never wake up*, etc., were extracted from the users’ discourse.

The *Linguistic Inquiry and Word Count* (LIWC; Pennebaker et al., 2001) and software (Welcome to LIWC-22, 2022) was used for extracting features for positive and negative emotions, anxiety, and personal concerns. The LIWC has a lexicon that is used to estimate the frequency of terms from several psychologically significant categories in text. The categories of interest representing personal concerns used in this study were: *death* (e.g., “*I’m now in stage 4 and would like to be able to discuss death without everyone freaking out and screaming doctors will figure it out*”); *school* (e.g., *My college roommate and I shared a room for the entire 5 years of school—he helped me out when I was sick*, *I was in his wedding*, *and we now chat on a daily basis*); *job* (e.g., [*…*] *I obtained a job immediately after treatment was finished*, *and I walked into the interview bald.*); *money* (e.g., *I owed $18*,*000 in addition to my $700 monthly payment for my first experience through cancer with a standard coverage* […]); *body* (e.g., *I want to live*, *yet my body seems to be attempting to murder me. My body’s latest surprise is colon cancer* […]); and *sexual* (e.g., *Returning to the subject of radiation*… “*I’m not sure I’d go through with it again.* […] *I’m a woman*, *which has its own set of negative consequences. I’m 40 and it’s messed up my sex life like nothing else*, *and menopause is no joke”*).

#### 2.3.2. Machine learning-based discourse analysis

We further explored the depression cues through an automatic model for detecting the depressive posts from the social media discourse of the users. The automatic model was trained on the dataset provided by Pirina and Çöltekin (2018). The dataset contains texts from Reddit and depression-related forums. Unlike other datasets for depression detection, this dataset also contains samples from individuals with a cancer diagnosis. It contains a total of 3,023 posts labeled as depression loaded and 3,058 posts labeled as non-depressed. For detecting the depression-loaded content, a transfer learning approach was chosen using a BERT model (Devlin et al., 2019), given its capacity to obtain state-of-the-art results on a wide variety of NLP tasks, including mental health problems detection (Matero et al., 2019; Martínez-Castaño et al., 2020). Transfer learning in NLP (Ruder et al., 2019) is a method in which the knowledge from a model that was pre-trained on general NLP tasks is transferred to downstream tasks, in our case, the task of detecting posts with depression cues. The pre-training performed by Devlin et al. (2019) consisted of two tasks: predicting masked target words and next sentence prediction from a large corpus of texts. The pre-trained BERT model was fine-tuned on the training data from Pirina and Çöltekin (2018) for one epoch with a learning rate of 0.00002 and Adam (Kingma and Ba, 2014; Martin and Johnson, 2015) optimizer with a linear decay scheduler. In order to evaluate the performance of the model, a *k*-fold cross-validation (*k* = 5) was performed. The dataset was split into five samples for cross-validation. For each of the five iterations, a different instance of the BERT model was trained on *k*-1 samples and evaluated on the remaining sample, this process was repeated for all the k folds of the data. *The model obtained an overall F1 score of 0.91*, *computed as the average of the F1 scores obtained for each fold.* After performing cross-validation and evaluating the model, a final BERT model was fine-tuned on all the data and used to automatically detect the posts with depression cues from the users in the current study. The F1-score was chosen to measure the performance of the model because it takes into account both precision and recall.

#### 2.3.3. Automatic topic modeling

**Figure 1:**
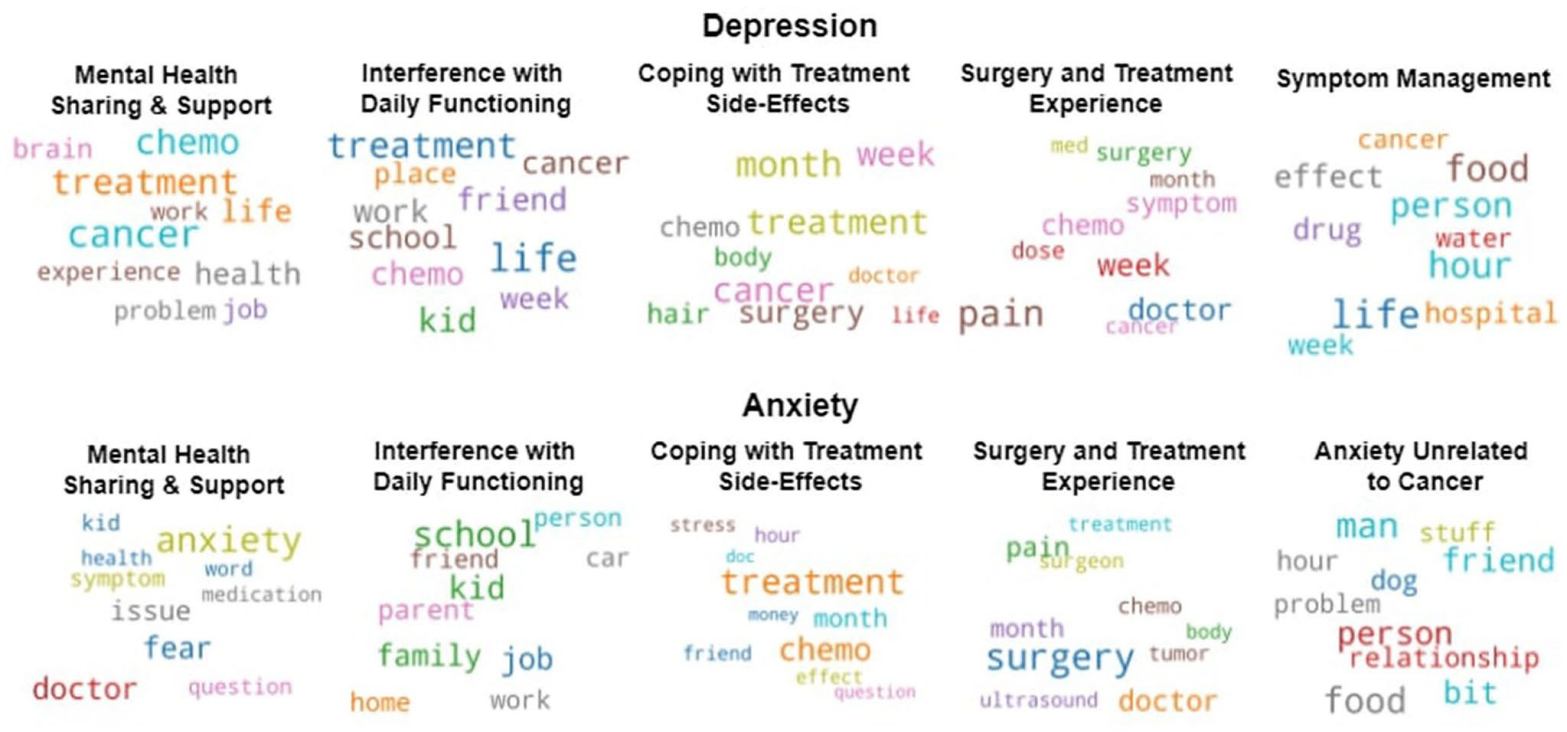
Top 10 most frequently occurring words per topics.

The depression and anxiety-loaded content was further explored to understand the topics that are discussed on Reddit by cancer survivors. The depression-loaded content is represented by posts labeled as containing depression cues by the automated model. The anxiety-loaded content consists of posts that contain at least one word from the anxiety category from LIWC. Two different Latent Dirichlet Allocation (LDA) models (Blei et al., 2001) were trained separately on the depression and anxiety-loaded posts. The model was trained using only the lemmatized nouns (transforming each word into its dictionary form) from the texts in the dataset in order to obtain a better performance, compared to the approach of training the model on the raw data (Martin and Johnson, 2015). Finally, a coherence score (measuring how interpretable topics are to humans; Röder et al., 2015; Burchett et al., 2017) was computed for a various number of topics ranging from 1 to 40 with increments of 5. *Five topics for depression* and *five topics for anxiety-loaded* content obtained the best coherence score. The topics from each category of content were labeled after analyzing text samples and the top 10 most frequently occurring words from all the topics (Figure 1).

Four topics are common to both depression and anxiety-loaded content. These are “Mental Health Sharing & Support,” “Interference with Daily Functioning,” “Coping with Treatment Side-Effects,” and the “Surgery and Treatment Experience” (see Tables 1, 2 for text samples). One other topic (i.e., “Symptom Management”) was specific to depression-loaded content, while “Anxiety unrelated to cancer” was a topic specific to anxiety-loaded content.

**Table 1:** Examples for each topic from the depression-loaded content.

Topic	Keywords	Example
Mental health sharing and support	Cancer, chemo, treatment, life, health, brain, job, problem, experience, and work	If I were you, I would try to see a psychiatrist […] and a counselor or therapist who practices CBT. With my depression, both types of treatment have been helpful.
Interference with daily functioning	Life, treatment, kid, chemo, work, school, friend, cancer, week, and place	[…] Because of depression and how much it affected my grades, I dropped out of graduate school today […]
Coping with treatment side-effects	Month, cancer, treatment, week, surgery, chemo, hair, body, life, and doctor	[…] I am a young woman who used to have long hair. […] After starting radiation, I ended up losing most of it anyway, so having my head shaved was a welcome respite… or maybe simply shave the section of the incision if you do not need therapy. […]
Surgery and treatment experience	Pain, week, doctor, chemo, symptom, surgery, dose, month, cancer, and med	[…] Everyone is different, but from my experience, I do not feel well for about 5 days after my treatment. I am fine on the first day, just tired. I have nausea, exhaustion, and a blah feeling on those days. I sleep a lot the first few days, then suffer from insomnia. […]
Symptom management	Life, hour, food, person, effect, drug, hospital, week, cancer, and water	Do you have discomfort when you eat? […] I am simply interested if anyone else has experienced this. If so, what aided you? I am feeling like shit. I cannot eat, drink, or sleep. […]

**Table 2:** Examples for each topic from the anxiety-loaded content.

Topic	Keywords	Example
Mental health sharing and support	Anxiety, doctor, fear, issue, symptom, question, kid, word, medication, and health	[…] It is possible that your thyroid hormones are worsening your existing depression. […] I use Xanax to treat my anxiety. I have never found antidepressants to be very helpful. Perhaps they can help you. The end of the tunnel is in sight. You will overcome this. I wish you fast mental recovery! […]
Interference with daily functioning	School, kid, job, family, parent, home, work, car, friend, and person	[…] Just thinking about going out, interviewing, and having to work in house gives me an anxiety attack. […] Then again, I just got diagnosed with cancer 2 months ago and need my health insurance. So I am trapped by choice and by circumstances
Coping with treatment side-effects	Treatment, chemo, month, hour, friend, effect, stress, question, money, and doc	Yes. I have completed my active treatment (for now). My only lingering problem is fatigue.
		If my body is too exhausted to move but my head is engaged and I cannot sleep, I consider it weariness. […]
		I can work a few hours per day, but I am always debating if the stress of not knowing if I will make my deadlines (due to fatigue) is greater than the stress / boredom of not working (I like work). […]
Surgery and treatment experience	Surgery, doctor, pain, month, chemo, tumor, surgeon, ultrasound, treatment, and body	[…] About a month following my operation, I began chemo. Results, scans, etc. take time to complete. I think this was the most anxious time because I was only awaiting the results to see if I required additional treatment or not. The removal surgery is not very difficult. […]
Anxiety unrelated to cancer	Man, food, person, friend, bit, dog, relationship, hour, stuff, and problem	After a night of drinking, I lost my phone and money. It took a couple of long, panicked hours for me to realize it had fallen between the bed and the nightstand and onto the floor.

### 2.4. Quantitative data analyses

The percentages of posts from each topic were computed for short-term, transition and long-term survivorship. Moreover, we conducted chi-square analyses for both depression and anxiety-charged content in order to investigate if there were any statistically significant differences between short, transitional, and long-term cancer survivors groups with regard to topic frequencies. In case of statistically significant results, the main analyses were followed by *post hoc* Bonferroni-adjusted *z*-tests.

The Shapiro–Wilk multivariate normality test indicated that the MANOVA assumption of normality was not met (W = 0.05, *p* = 0.001). For this reason, we adopted a nonparametric inference approach for multivariate data (“npmv” R package; Kingma and Ba, 2014). This approach allows for the calculation of a multivariate global test of statistical significance, which can then be followed by univariate nonparametric analyses. We used the Muller approximation for the Bartlett Nanda Pillai type indicator (Harrar and Bathke, 2008) for the global test of significance, the equivalent of MANOVA’s Pillai’s trace, as recommended when the sample sizes are unequal (Field, 2013), with 1,000 permutations. The global test of significance was followed by univariate Kruskal-Wallis tests, which employed Bonferroni corrections for multiple pairwise comparisons between cancer survivor groups.

### 2.5. Qualitative data analyses

Regarding suicide posts, we wanted to supplement the quantitative analysis with more details about the content of Reddit posts and comments from cancer survivors. Topic modeling was not appropriate for this goal because we had few texts labeled as suicide-related (*N*  = 260), far fewer than the texts on anxiety (*N* = 5,405) and depression (*N* = 14,558) for which topic modeling was performed.

We manually followed a two-step procedure (for a review see Thomas, 2006; Soiferman, 2010). First, the main themes (categories) were established before manual annotations based on previous literature (Coppersmith et al., 2018; Ji et al., 2018) and our observations on the text. Second, the text labeling was performed by two annotators (DT and AL) under the supervision of the main author (IRP). After individually rating the dataset—with an excellent interrater agreement (Cohen’s *κ* = 0.93)—the remaining differences were further discussed with the supervisor until total agreement was met.

The final categories consisted of the following topics: (1) *expressing past or present suicidal thoughts/attempts* (i.e., “*Okay*, *I’m going to commit suicide”*), (2) *offering support and formally discussing about suicide* (educational support, referring to other’s suicide; i.e., “*Nobody can stop a person who has decided that they want to die. My grandfather told me this after a close friend of mine committed suicide”*), and (3) texts *not relevant to suicide* (i.e., “*So*, *Suicide Squad is no longer canon?”*).

## 3. Results

### 3.1. Sample characteristics

The cancer survivors ranged in age from 18 to 73 years old (M = 36.12, SD = 11.14), with most of the participants being female (52.40%), in a significant relationship or married (55.10%), and employed (67.4%). A little more than half the participants (54%) were undergoing cancer treatment at the time of data collection.

With regard to cancer type, of the 12 types of cancer that participants reported being diagnosed with, the most frequent were lymphoma/leukemia (19.80%), testicular cancer (16.60%), and breast cancer (16%). Seven participants reported other, more rare types of cancer, such as bladder cancer, adrenal cancer, or medullary aplasia. The majority of the participants reported that they did not experience a cancer recurrence episode (83.40%). With regard to comorbidities, 45.50% of participants reported having a psychiatric diagnosis, while 36.40% had a chronic disease. All stages of cancer were reported, with most of the participants reporting early stages of cancer (I, II; 56.70%), followed by stage III and IV cancer (31.60%), while 22 participants (11.8%) reported no stage at diagnosis.

With regard to cancer treatment, the participants reported a wide range of standalone or combinations of treatments (Ntreatment = 30), with surgery (21.40%), chemotherapy (11.80%), chemotherapy + surgery (15%), and chemotherapy + surgery + radiotherapy (11.20%) being the most frequently employed.

### 3.2. Quantitative analysis

The nonparametric multivariate analysis revealed a statistically global difference between groups, with the Muller approximation for the Bartlett-Nanda-Pillai test being *F* (28.44; 347.58) = 1.88, *p* = 0.005. As such, we continued with the separate univariate Kruskall-Wallis tests and the *post hoc* pairwise comparisons (Table 3), both rendered below. Summing up in advance, the majority of the *post hoc* pairwise comparisons found significant differences between short-term and long-term cancer survivors, with higher frequencies in the short-term cancer survivors group.

**Table 3:** *Post hoc* pairwise comparisons between cancer survivors’ groups.

	Short-term vs. Transition	Long-term vs. Transition	Long-term vs. Short-term
ML-classified depression posts	U = 21.87, *p*^a^ = 0.066	U = −13.25, *p* = 0.597	U = −35.13, *p* = 0.001
Anxiety-related terms	U = 15.30, *p* = 0.327	U = −10.80, *p* = 0.886	U = −26.10, *p* = 0.019
Number of posts	U = 0.27, *p* = 1.000	U = −26.63, *p* = 0.030	U = −26.36, *p* = 0.017
Cancer-related terms	U = 16.28, *p* = 0.265	U = −28.45, *p* = 0.018	U = −44.73, *p* < 0.001
Death-related terms	U = −11.11, *p* = 0.729	U = 13.83, *p* = 0.536	U = −24.95, *p* = 0.026
School-related terms	U = −16.55, *p* = 0.249	U = 9.60, p = 1.000	U = −26.16, *p* = 0.018
Money-related terms	U = −25.43, *p* = 0.023	U = −10.78, *p* = 0.888	U = 14.65, *p* = 0.375
Body-related terms	U = 13.18, *p* = 0.503	U = −20.31, *p* = 0.147	U = −33.50, *p* = 0.001

^a^Significance values have been adjusted by the Bonferroni correction for multiple tests.

#### 3.2.1. Depression-loaded content

With regard to *ML-classified depression* posts, the Univariate Kruskal-Wallis test was statistically significant—H(2) = 14.21, *p* = 0.001—and *post hoc* analyses revealed statistically significant differences between short-term and long-term groups in terms of the number of machine-learning classified depression posts (Table 3). Short-term cancer survivors had a higher frequency of depression-related posts than long-term cancer survivors (*p* = 0.001).

**Figure 2:**
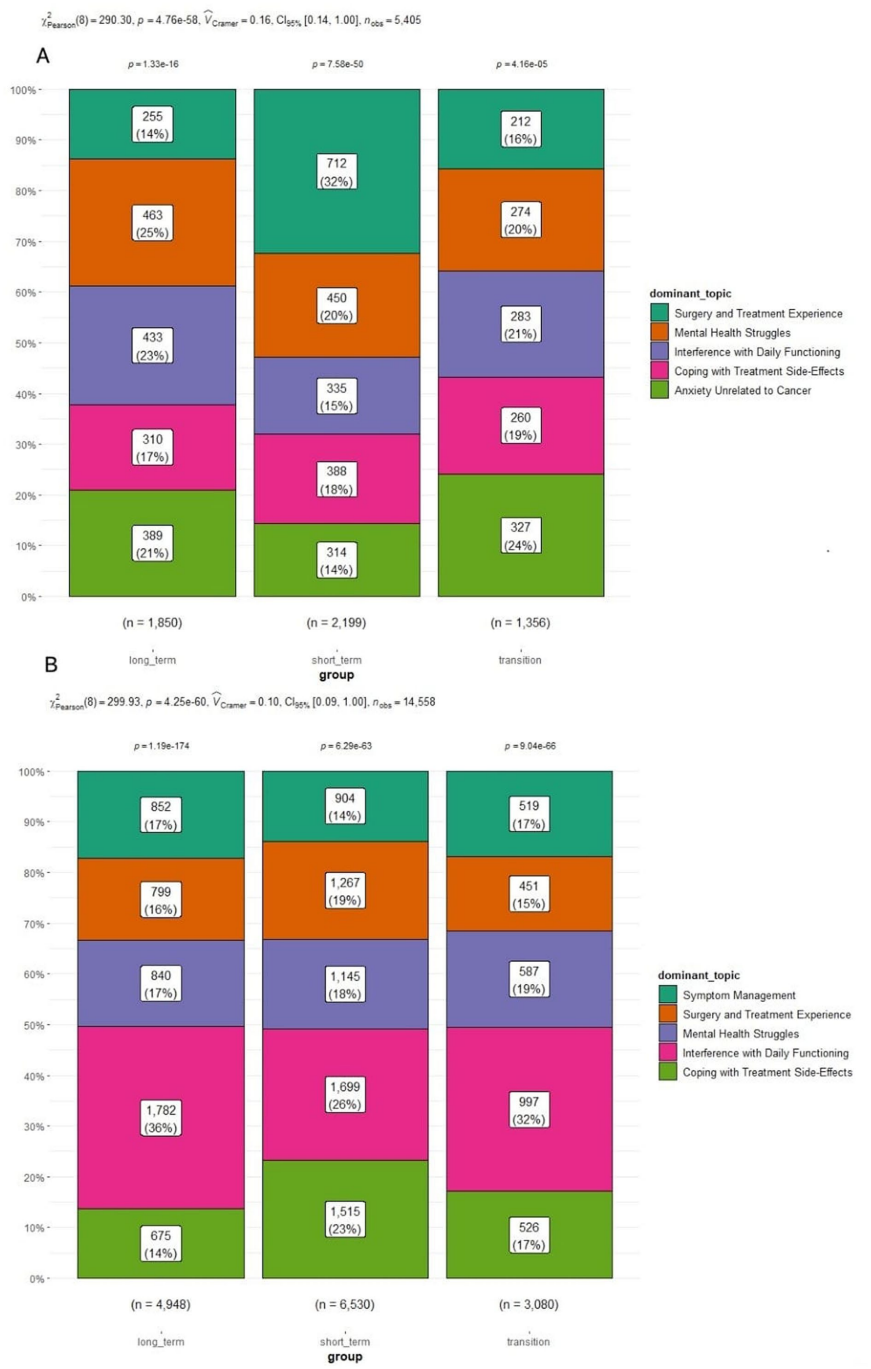
Main topics per survivorship group for **(A)** anxiety-loaded words and **(B)** depression-loaded posts.

With regard to topic modeling of depression-loaded content, the association between cancer survivor groups and topic was statistically significant, *χ*^2^ (8, *N* = 14,558) = 299.93, *p* < 0.001 (Figure 2). The most visibly prevalent topic across all stages of survivorship was “*Interference with Daily Functioning.”* The *post hoc* analysis revealed that all pairwise differences between cancer survivor groups were statistically significant (*p* < 0.05), with greater frequencies being observed in the long-term group (36%) than in the short-term (26%) or transition (32.4%) groups. All pairwise differences were significant (*p* < 0.05) also in the case of the topic “*Coping with Treatment Side-Effects*,*”* greater post frequencies being observed in the short-term group (23.2%) than in the long-term (13.6%) or transition (17.1%) groups.

In the case of the “*Surgery and Treatment Experience*,” the *post hoc* analysis revealed that there were statistically significant (*p* < 0.05) differences between short-term (19.4%) and long-term (16.1%) or between short-term and transition (14.6%) groups. “*Symptom Management*” wise, the *post hoc* analysis revealed that there were statistically significant (*p* < 0.05) differences in terms of post frequencies between short-term (13.8%) and long-term (17.2%) or between short-term and transition (16.9%) groups. There were no statistically significant differences between groups for the “*Mental Health Sharing & Support*” topic.

#### 3.2.2. Anxiety and suicide-related terms

For *anxiety-related terms*, the Univariate Kruskal-Wallis test was statistically significant—H(2) = 7.73, *p* = 0.021 (Figure 2) and *post hoc* analyses revealed statistically significant differences between short-term and long-term groups (Table 3). Short-term cancer survivors had a higher frequency of anxiety-related terms across their posts than long-term cancer survivors (*p* = 0.019).

Regarding topic modeling of anxiety-loaded content, the association between cancer survivor groups and topic was statistically significant, χ^2^ (8, *N* = 5,405) = 290.30, *p* < 0.001. For the topic “*Surgery and Treatment Experience*,” in which users discuss their anxiety related to the surgery and cancer treatment, the *post hoc* analysis revealed that there were statistically significant (*p* < 0.05) differences in terms of post frequencies between short-term (32.4%) and long-term (13.8%) or short-term and transition (15.6%) groups. For *“Interference with Daily Functioning*,” the *post hoc* analysis revealed that there were statistically significant (*p* < 0.05) differences in terms of post frequencies between short-term (15.2%) and long-term (23.4%) or short-term and transition (20.9%) groups.

The “*Mental Health Sharing & Support*” topic revealed statistically significant (*p* < 0.05) differences in terms of post frequencies between long-term (25%) and short-term (20.5%) or long-term and transition (20.2%) groups. The “*Anxiety Unrelated to Cancer*” recorded statistically significant (*p* < 0.05) differences in terms of post frequencies between short-term (14.3%) and long-term (21%) and between short-term and transition (24.1%) groups. There were no statistically significant differences between groups for the “*Coping with Treatment Side-Effects*” topic.

Regarding *suicide-related terms*, the Univariate Kruskal-Wallis test was statistically non-significant—H(2) = 4.01, *p* = 0.134 (Table 3). Nevertheless, we think it is instructive to examine the posts that were identified as being about suicide. The majority of these posts (59.20%) dealt with providing assistance or openly talking about suicide (for example, by mentioning the suicide of others or providing educational support). Quite a significant percentage of posts (11.50%) consisted of expressing past or present suicidal thoughts or attempts. Finally, 29.20% of posts were not relevant in terms of suicide-related terms.

#### 3.2.3. Personal concerns and cancer-related terms

For cancer-related terms, the Univariate Kruskal-Wallis test was statistically significant—H(2) = 21.99, *p* < 0.001 and *post hoc* analyses revealed statistically significant differences between the short-term and long-term groups, and transition vs. long-term groups (Table 3). Short-term cancer survivors had a higher frequency of cancer-related terms across their posts than the survivors from the long-term group (*p* < 0.001). Moreover, transition cancer survivors had a higher frequency of cancer-related terms across their posts than the survivors from the long-term group (*p* = 0.018).

For *death-related terms*, the Univariate Kruskal-Wallis test was statistically significant—H(2) = 6.87, *p* = 0.032 and *post hoc* analyses revealed statistically significant differences between short-term and long-term groups (Table 3). Short-term cancer survivors had a higher frequency of death-related terms across their posts than long-term cancer survivors (*p* = 0.026).

For *school-related terms*, the Univariate Kruskal-Wallis test was statistically significant—H(2) = 6.87, *p* = 0.032, and *post hoc* analyses revealed statistically significant differences between short-term and long-term groups (Table 3). Short-term cancer survivors had a higher frequency of school-related terms across their posts than long-term cancer survivors (*p* = 0.018).

For *money-related terms*, the Univariate Kruskal-Wallis test was statistically significant—H(2) = 7.30, *p* = 0.026 and *post hoc* analyses revealed statistically significant differences between short-term and transition groups. (Table 3). Transition cancer survivors had a higher frequency of money-related terms across their posts than short-term cancer survivors (*p* = 0.023).

For *body-related terms*, the Univariate Kruskal-Wallis test was statistically significant—H(2) = 12.30, *p* = 0.002 and *post hoc* analyses revealed statistically significant differences between short-term and long-term groups. (Table 3). Short-term cancer survivors had a higher frequency of body-related terms across their posts than long-term cancer survivors (*p* = 0.001).

No statistically significant group effects were observed for positive [H(2) = 1.84, *p* = 0.397] or negative emotions-related terms [H(2) = 1.45, *p* = 0.482], and for job [H(2) = 0.92, *p* = 0.629] or sexual-related terms [H(2) = 2.33, *p* = 0.311].

We also looked into differences concerning the number of words and the number of posts. There were no differences regarding the number of words. However, there were significant differences in regard to the number of posts, H(2) = 9.29, *p* = 0.010. *Post hoc* analyses revealed that both short-term (*p* = 0.017) and transitioning (*p* = 0.030) cancer survivors had significantly more posts than long-term cancer survivors.

## 4. Discussion

The current study aimed to develop a comprehensive picture of cancer survivors’ mental health in online environments. For this purpose, cancer survivors’ Reddit discourse was analyzed at three relevant time points, namely short-term (0–2 years post-diagnosis), transition (> 2–4 years post-diagnosis), and long-term (≥ 5 years post-diagnosis; Götze et al., 2014; Okado et al., 2016; Wang et al., 2016).

The results showed a consistent pattern for the main outcomes. Short-term survivors used significantly more depression-loaded posts and anxiety-loaded words than long-term survivors, with no significant differences relative to the transition period. *From a theoretical perspective*, these findings are supported by the *diathesis-stress model* (Zubin and Spring, 1977; Monroe and Simons, 1991; Zuckerman, 1999) and the *cancer survivor adaptation model* (CSA; Naus et al., 2009). Recently, Lee and Jeong (2019) supported the applicability of this latter theoretical model by showing that adaptation mediates the relation between the components of the CSA model and quality of life in cancer patients. Symptoms may accentuate in the presence of the stressor and subside as the stressor becomes distal. Short-term cancer survivors may be vigilant due to the higher risk of cancer recurrence and gradually adjust once the major hurdles are overcome.

*From an empirical perspective*, the results are supported by studies showing a decrease in anxiety (Williams et al., 2021) and depression (Schroevers et al., 2006; Mols et al., 2018) in cancer patients, contrary to research showing an increase in psychological symptoms in the long-term compared to the short-term survivorship (Breidenbach et al., 2022). The similar pattern in the results for anxiety and depression is also in line with the well-known comorbidity between the two mental health conditions, which has also been studied in cancer patients (Brown et al., 2010).

The topic analysis evidenced commonalities between depression and anxiety-labeled texts such as posts regarding “Mental Health Sharing & Support,” symptom “Interference with Daily Functioning,” as well as the manifold ways of “Coping with Treatment Side-Effects,” and insights about the “Surgery and Treatment Experience.” However, some differences between anxiety and depression labeled texts emerged (i.e., “Symptom Management” for depression-loaded posts and “Anxiety Unrelated to Cancer” for anxiety-loaded posts).

These topics reflect the stressors and the variations in concerns depending on different stages of survivorship. Short-term survivors discussed more about the topics concerning their experience related to surgeries and treatment and their ways of coping with side effects. Long-term cancer survivors shared more about their struggles with mental health issues and offered support to other users (i.e., anxiety-loaded posts), as well as discussed how cancer interfered with their daily functioning. Such topic examples are provided in Tables 1, 2. Additionally, unlike short-term survivors, transition and long-term survivors discussed their anxiety unrelated to cancer (i.e., anxiety-loaded posts). These results suggest that cancer disrupts survivors’ daily functioning despite an improvement in their psychological state in the long run. Similarly, research indicated that although mental-health related outcomes either remained constant or decreased, cancer survivors tend to report an increase in physical symptoms from 15 months to even 8 years following diagnosis (Schroevers et al., 2006). The mostly non-significant differences in the transition phase are somewhat to be expected based on a continuum of adjustment to a new normality.

Suicide is an important correlate of mental health. In the current study, the frequency of suicide-related words was relatively constant between the survivorship categories. Most of the suicide-related posts referred to offering support or discussing formally about suicide, while suicide thoughts and past or present attempts have been mentioned to a lesser extent. This is consistent with the fact that Reddit is by design meant to encourage social support (Choudhury and De, 2014) and became a source of support for coping with suicidal ideation during the COVID-19 pandemic (McAuliffe et al., 2022). However, the low number of submissions labeled as suicide-related precluded us from drawing other conclusions.

In terms of personal concerns, short-term cancer survivors used more words related to death, school, and body compared to long-term cancer survivors, but fewer words related to money compared to transitioning cancer survivors. In addition, long-term cancer survivors had fewer posts and cancer-related words overall than short-term and transition cancer survivors, although “cancer” remained one of the most prevalent words used for all three categories, suggesting the strong impact around the time of diagnosis but also the pervasiveness of cancer as a lifelong disease. The frequency of the terms related to job and sexuality and the terms related to positive and negative emotions did not differ across categories.

The study has several limitations worth mentioning. *First*, a maximum of 1,000 submissions and comments were gathered from each user because of the limitations of the PRAW library. However, we did succeed in collecting all the posts from the majority of the cancer survivors, which were usually less than 1,000, and the risk of missing out on relevant information was mitigated. *Second*, the lexicon-based approach used for several of the analyzed features was a simple unsupervised method, relying on a predefined list of words (e.g., LIWC dictionary), which are identified regardless of the context in which the words from the lexicons were found. *Third*, some markers of depression may also be treatment side-effects, such as fatigue and low energy (Ma et al., 2020). Therefore, there may be a risk of over-identifying the linguistic markers of depression. However, this is a well-known limitation of self-reports as well (Brown et al., 2010), and NLP techniques showed acceptable performance of automated algorithms for classifying depression (e.g., Guntuku et al., 2017; Ruba and Diana, 2020). *Fourth*, a limitation of our study is the lack of precise information on the year of psychiatric diagnosis and recovery for each participant. This temporal data would have been valuable in aligning mental health diagnoses with social media posts. However, since not all psychiatric diagnoses would have been relevant to our study’s focus, it would have been difficult to establish such alignments. Additionally, relying on participants’ recall of diagnosis and recovery timing could introduce variability, particularly for those diagnosed long ago. Future studies should prioritize collecting data on diagnosis and remission timelines to improve the clinical relevance of their findings. *Fifth*, even though this type of study has obtained high-quality data straight from the source and only a few such studies ask for permission to extract publicly available data, a relatively small number of survey respondents gave us permission to use their data. Future studies could gather data from multiple social media streams to expand their data set. *Sixth*, the latter limitation is rather a disclaimer. Though this is a clinically relevant study, it is not a clinical diagnosis or standard screening research, and no claims of such nature are made. To better approach such a type of research, future studies should also collect the scores from screening questionnaires alongside a clinical diagnosis of their volunteers.

Despite these drawbacks, our study has several advantages and implications. To our knowledge, this is the first study to investigate the mental health of cancer survivors using NLP techniques depending on their stages of survivorship. A major advantage of this research is the authenticity of the data collected from Reddit. Text analysis offered the possibility of accessing participants’ experiences through their discourse, beyond simple recollection. Moreover, in the current study, the year of diagnosis was provided by the participants; therefore, grouping posts into the three categories was based on precise information, providing better insight.

This study has clinical ramifications. Before speaking with a mental health professional, many cancer survivors turn to social media platforms like Reddit to express their feelings and seek support from other survivors (Hartzler and Pratt, 2011; Tripathi et al., 2022). This sets the stage for Reddit to become a platform for screening and prevention. Automated methods embedded within Reddit can serve as the initial component of a stepped care model by identifying survivors at risk for or experiencing mental health issues and referring them to a specialist for further diagnosis. Moreover, by combining traditional screening methods with social media insights, it is possible to gain a more complete understanding of the mental health of survivors.

This study also underscores the importance of recognizing the unique experiences and challenges faced by cancer survivors at different stages of their survivorship journey. The topic analyses conducted in this study revealed specific obstacles encountered by survivors, including difficulties with daily functioning, treatment side effects, and surgical experiences. This valuable information can inform the development of targeted interventions and support programs tailored to the specific needs of survivors at various stages. The results also suggest that short-term cancer survivors may require focused interventions to address their signs of depression and anxiety, while long-term survivors may benefit from ongoing support to manage the enduring impact of cancer on their lives.

Ultimately, the linguistic patterns identified here should be corroborated by future NLP studies and studied further in clinical settings that include formal diagnoses.

### 4.1. Conclusion

This research utilizes authentic data from Reddit to contribute to the understanding of mental health throughout the survivorship stages. Analysis of textual content reveals that depressive and anxious posts are most prevalent during the short-term survival period. Short-term survivors also express more concerns about death, cancer interfering with education, and several body-related issues. Long-term cancer survivors, on the other hand, have fewer depression and anxiety-loaded posts but continue to mention “cancer,” though to a lesser extent than short-term survivors, emphasizing its enduring impact. They also seem to have the resources necessary to share their prior experiences with suicidal ideation and mental health struggles while providing support to their survivor community.

The topic analyses conducted in this study shed light on specific challenges faced by cancer survivors, such as difficulties with daily functioning, treatment side effects, and surgical experiences. This valuable information can guide the development of targeted interventions and support programs that cater to the needs of survivors at different stages. Furthermore, the findings indicate that short-term cancer survivors may benefit from focused interventions to address signs of depression and anxiety, while long-term survivors may require support to cope with the impact of cancer on their lives.

Overall, this study highlights the importance of utilizing text-based content to access first-hand accounts and timely recognition of active stressors and mental health triggers in the context of cancer survivorship. This approach provides valuable insights and a more comprehensive understanding of the mental health of survivors. Future studies are needed on samples that include a formal diagnosis.

## Data availability statement

The datasets presented in this study can be found in online repositories. The names of the repository/repositories and accession number(s) can be found at: https://osf.io/7bcte/?view_only=e46cec39d29e4049ba112c12cf92805a.

## Ethics statement

The studies involving human participants were reviewed and approved by the Research Committee of the University of Bucharest. The patients/participants provided their written informed consent to participate in this study.

## Author contributions

IRP designed and coordinated the study. Material preparation and data collection were performed by IRP and DT, and data analysis by A-MB and LF with the contribution of DT and AL. LD and RFB supervised the natural language processing analyses. The first draft of the manuscript was written by IRP, A-MB, AL, and LF with the contribution of DT, LD, and RFB. IRP revised and supervised the entire manuscript. All authors contributed to the article and approved the submitted version.

## Funding

This work was supported by a grant of the Romanian Ministry of Education and Research, CNCS-UEFISCDI, project number PN-III-P1-1.1-TE-2019-2140, within PNCDI III. The funding entity was not involved in any matter in the data collection, results or any stage of the research.

## Conflict of interest

The authors declare that the research was conducted in the absence of any commercial or financial relationships that could be construed as a potential conflict of interest.

## Publisher’s note

All claims expressed in this article are solely those of the authors and do not necessarily represent those of their affiliated organizations, or those of the publisher, the editors and the reviewers. Any product that may be evaluated in this article, or claim that may be made by its manufacturer, is not guaranteed or endorsed by the publisher.

## References

[ref1] Ammari T. Schoenebeck S. Romero D. (2019). Self-declared throwaway accounts on Reddit: how platform affordances and shared norms enable parenting disclosure and support. Proc. ACM Hum. Comput. Interact. 3, 1–30. doi: 10.1145/335923734322658

[ref2] Bergerot C. D. Mitchell H.-R. Ashing K. T. Kim Y. (2017). A prospective study of changes in anxiety, depression, and problems in living during chemotherapy treatments: effects of age and gender. Support Care Cancer 25, 1897–1904. doi: 10.1007/s00520-017-3596-9, PMID: 28150043

[ref3] Blei D. Ng A. Jordan M. (2001). Latent Dirichlet allocation. J. Mach. Learn. Res. 3:608.

[ref42] Boe B. (2016). PRAW The Python Reddit API Wrapper. Available at: https://github.com/praw-dev/praw

[ref4] Breidenbach C. Heidkamp P. Hiltrop K. Pfaff H. Enders A. Ernstmann N.. (2022). Prevalence and determinants of anxiety and depression in long-term breast cancer survivors. BMC Psychiatry 22:101. doi: 10.1186/s12888-022-03735-3, PMID: 35139815 PMC8827186

[ref5] Brown L. F. Kroenke K. Theobald D. E. Wu J. Tu W. (2010). The association of depression and anxiety with health-related quality of life in cancer patients with depression and/or pain. Psychooncology 19, 734–741. doi: 10.1002/pon.1627, PMID: 19777535 PMC2888919

[ref6] Bucur A.-M. Dinu L. P. (2020). “Detecting early onset of depression from social media text using learned confidence scores”, in Proceedings of the Seventh Italian Conference on Computational Linguistics, 73–78.

[ref7] Bucur A.-M. Podină I. R. Dinu L. P. (2021). “A psychologically informed part-of-speech analysis of depression in social media”, Proceedings of the International Conference on Recent Advances in Natural Language Processing, 199–207. doi: 10.26615/978-954-452-072-4_024

[ref8] Burchett W. W. Ellis A. R. Harrar S. W. Bathke A. C. (2017). Nonparametric inference for multivariate data: the R package npmv. J. Stat. Softw. 76, 1–18. doi: 10.18637/jss.v076.i0436568334

[ref9] Calvo R. A. Milne D. N. Hussain M. S. Christensen H. (2017). Natural language processing in mental health applications using non-clinical texts†. Nat. Lang. Eng. 23, 649–685. doi: 10.1017/S1351324916000383

[ref10] Carreira H. Williams R. Müller M. Harewood R. Stanway S. Bhaskaran K. (2018). Associations between breast cancer survivorship and adverse mental health outcomes: a systematic review. J. Natl. Cancer Inst. 110, 1311–1327. doi: 10.1093/jnci/djy177, PMID: 30403799 PMC6292797

[ref11] Choudhury M. D. De S. “Mental health discourse on reddit: self-disclosure, social support, and anonymity” in *Eighth International AAAI Conference on Weblogs and Social Media* (2014).

[ref12] Choudhury M. D. Gamon M. Counts S. Horvitz E. (2013). Predicting depression via social media. Proc. Int. AAAI Conf. Web Soc. Media 7, 128–137. doi: 10.1609/icwsm.v7i1.14432

[ref13] Coppersmith G. Leary R. Crutchley P. Fine A. (2018). Natural language processing of social media as screening for suicide risk. Biomed. Inform. Insights 10:117822261879286. doi: 10.1177/1178222618792860 PMC6111391 30158822

[ref14] Crossley S. Paquette L. Dascalu M. McNamara D. S. Baker R. S. “Combining click-stream data with NLP tools to better understand MOOC completion” in *Proceedings of the Sixth International Conference on Learning Analytics & Knowledge*. Association for Computing Machinery, 6–14. (2016).

[ref15] Devlin J. Chang M.-W. Lee K. Toutanova K. (2019). “BERT: pre-training of deep bidirectional transformers for language understanding”, in Proceedings of the 2019 Conference of the North American Chapter of the Association for Computational Linguistics: Human Language Technologies, 1, 4171–4186. doi: 10.18653/v1/N19-1423

[ref16] Field A. (2013). Discovering Statistics Using IBM SPSS Statistics. And Sex and Drugs and Rock “N” Roll, 4th Edition, Los Angeles, London:SAGE.

[ref17] Free Online Survey Software and Tools|QuestionPro® (n.d.). FREE Online Surveys. Available at: https://www.questionpro.com/

[ref18] Götze H. Brähler E. Gansera L. Polze N. Köhler N. (2014). Psychological distress and quality of life of palliative cancer patients and their caring relatives during home care. Support Care Cancer 22, 2775–2782. doi: 10.1007/s00520-014-2257-5, PMID: 24811216

[ref19] Guntuku S. C. Yaden D. B. Kern M. L. Ungar L. H. Eichstaedt J. C. (2017). Detecting depression and mental illness on social media: an integrative review. Curr. Opin. Behav. Sci. 18, 43–49. doi: 10.1016/j.cobeha.2017.07.005

[ref20] Harrar S. W. Bathke A. C. (2008). A nonparametric version of the Bartlett-Nanda-Pillai multivariate test. Asymptotics, approximations, and applications. Am. J. Math. Manag. Sci. 28, 309–335. doi: 10.1080/01966324.2008.10737731

[ref21] Hartzler A. Pratt W. (2011). Managing the personal side of health: how patient expertise differs from the expertise of clinicians. J. Med. Internet Res. 13:e1728. doi: 10.2196/jmir.1728 PMC3222167 21846635

[ref22] Haun M. W. Sklenarova H. Brechtel A. Herzog W. Hartmann M. (2014). Distress in Cancer patients and their caregivers and association with the caregivers’ perception of dyadic communication. Oncol. Res. Treat. 37, 384–388. doi: 10.1159/000364885, PMID: 25138298

[ref23] Ji S. Yu C. P. Fung S. Pan S. Long G. (2018). Supervised learning for suicidal ideation detection in online user content. Complexity 2018:e6157249. doi: 10.1155/2018/6157249

[ref24] Jung E. Jain H. Sinha A. P. Gaudioso C. (2021). Building a specialized lexicon for breast cancer clinical trial subject eligibility analysis. Health Inform. J. 27:146045822198939. doi: 10.1177/1460458221989392, PMID: 33535885

[ref25] Kingma D. P. Ba J. (2014). “Adam: a method for stochastic optimization”, in Proceedings of the International Conference on Learning Representations, doi: 10.48550/arXiv.1412.6980

[ref26] Kuba K. Esser P. Mehnert A. Hinz A. Johansen C. Lordick F.. (2019). Risk for depression and anxiety in Long-term survivors of hematologic Cancer. Health Psychol. 38, 187–195. doi: 10.1037/hea0000713, PMID: 30762398

[ref27] Lee J. L. Jeong Y. (2019). Quality of life in patients with non–small cell lung Cancer: structural equation modeling. Cancer Nurs. 42, 475–483. doi: 10.1097/NCC.0000000000000645 30204597

[ref28] Ma Y. He B. Jiang M. Yang Y. Wang C. Huang C.. (2020). Prevalence and risk factors of cancer-related fatigue: a systematic review and meta-analysis. Int. J. Nurs. Stud. 111:103707. doi: 10.1016/j.ijnurstu.2020.103707, PMID: 32920423

[ref29] Mahvi D. A. Liu R. Grinstaff M. W. Colson Y. L. Raut C. P. (2018). Local Cancer recurrence: the realities, challenges, and opportunities for new therapies. CA Cancer J. Clin. 68, 488–505. doi: 10.3322/caac.21498, PMID: 30328620 PMC6239861

[ref30] Martin F. Johnson M. “More efficient topic Modelling through a noun only approach” in Proceedings of the Australasian Language Technology Association Workshop. (2015) 111–115.

[ref31] Martínez-Castaño R. Htait A. Azzopardi L. Moshfeghi Y. (2020). Early risk detection of self-harm and depression severity using BERT-based transformers: early risk prediction on the internet. CEUR Workshop Proc. 2696

[ref32] Matero M. Idnani A. Son Y. Giorgi S. Vu H. Zamani M.. (2019). “Suicide risk assessment with multi-level dual-context language and BERT” in *Proceedings of the Sixth Workshop on Computational Linguistics and Clinical Psychology*. 39–44. Association for Computational Linguistics.

[ref33] McAuliffe C. Slemon A. Goodyear T. McGuinness L. Shaffer E. Jenkins E. K. (2022). Connectedness in the time of COVID-19: Reddit as a source of support for coping with suicidal thinking. SSM Qual. Res. Health 2:100062. doi: 10.1016/j.ssmqr.2022.100062, PMID: 35224533 PMC8856747

[ref34] Mols F. Schoormans D. de Hingh I. Oerlemans S. Husson O. (2018). Symptoms of anxiety and depression among colorectal cancer survivors from the population-based, longitudinal PROFILES registry: prevalence, predictors, and impact on quality of life. Cancer 124, 2621–2628. doi: 10.1002/cncr.31369, PMID: 29624635 PMC6033166

[ref35] Monroe S. M. Simons A. D. (1991). Diathesis-stress theories in the context of life stress research: implications for the depressive disorders. Psychol. Bull. 110, 406–425. doi: 10.1037/0033-2909.110.3.406, PMID: 1758917

[ref36] Naughton M. J. Weaver K. E. (2014). Physical and mental health among Cancer survivors. N. C. Med. J. 75, 283–286. doi: 10.18043/ncm.75.4.283, PMID: 25046097 PMC4503227

[ref37] Naus M. J. Ishler M. D. Parrott C. E. Kovacs S. A. (2009). Cancer survivor adaptation model: conceptualizing cancer as a chronic illness. J. Clin. Psychol. 65, 1350–1359. doi: 10.1002/jclp.20622, PMID: 19827115

[ref38] Newman M. W. Lauterbach D. Munson S. A. Resnick P. Morris M. E. “It’s not that i don’t have problems, i’m just not putting them on facebook: challenges and opportunities in using online social networks for health.” in *Proceedings of the ACM 2011 conference on Computer supported cooperative work*. 341–350. Association for Computing Machinery (2011).

[ref39] Okado Y. Tillery R. Howard Sharp K. Long A. M. Phipps S. (2016). Effects of time since diagnosis on the association between parent and child distress in families with pediatric cancer. Child. Health Care 45, 303–322. doi: 10.1080/02739615.2014.996883, PMID: 27630380 PMC5019566

[ref40] Pennebaker J. Francis M. Booth R. Linguistic Inquiry and Word Count (LIWC). Mahwah, NJ: Lawrence Erlbaum Associates (2001).

[ref41] Pirina I. Çöltekin Ç. “Identifying depression on Reddit: the effect of training data” in *Proceedings of the 2018 EMNLP Workshop SMM4H: The 3rd Social Media Mining for Health Applications Workshop & Shared Task*. Association for Computational Linguistics, 9–12. (2018).

[ref43] Röder M. Both A. Hinneburg A. “Exploring the space of topic coherence measures” in *Proceedings of the Eighth ACM International Conference on Web Search and Data Mining*. Association for Computing Machinery, 399–408. (2015).

[ref44] Ruba S. Diana I. (2020). Using social Media for Mental Health Surveillance. ACM Comput. Surv. CSUR 53, 1–31. doi: 10.1145/3422824

[ref45] Ruder S. Peters M. E. Swayamdipta S. Wolf T. “Transfer learning in natural language processing” in *Proceedings of the 2019 Conference of the North American Chapter of the Association for Computational Linguistics: Tutorials*. Association for Computational Linguistics, 15–18 (2019).

[ref46] Sacerdoti R. C. Laganà L. Koopman C. (2010). Altered sexuality and body image after gynecological cancer treatment: how can psychologists help? Prof. Psychol. 41, 533–540. doi: 10.1037/a0021428, PMID: 21572538 PMC3092554

[ref47] Sawhney R. Manchanda P. Mathur P. Shah R. Singh R. “Exploring and learning suicidal ideation connotations on social media with deep learning” in *Proceedings of the 9th Workshop on Computational Approaches to Subjectivity*, *Sentiment and Social Media Analysis*. Association for Computational Linguistics, 167–175 (2018).

[ref48] Schroevers M. Ranchor A. V. Sanderman R. (2006). Adjustment to cancer in the 8 years following diagnosis: a longitudinal study comparing cancer survivors with healthy individuals. Soc. Sci. Med. 63, 598–610. doi: 10.1016/j.socscimed.2006.02.008, PMID: 16597479

[ref49] Segerstrom S. C. O’Connor D. B. (2012). Stress, health and illness: four challenges for the future. Psychol. Health 27, 128–140. doi: 10.1080/08870446.2012.659516 22348326

[ref50] Sekulic I. Gjurković M. Šnajder J. (2018). “Not just depressed: bipolar disorder prediction on Reddit.” in *Proceedings of the 9th Workshop on Computational Approaches to Subjectivity*, *Sentiment and Social Media Analysis*. Association for Computational Linguistics, 72–78.

[ref51] Shankar A. Prasad N. Roy Sh Chakraborty A. Biswas ASh Patil J.. (2017). Sexual dysfunction in females after Cancer treatment: an unresolved issue. Asian Pac. J. Cancer Prev. 18, 1177–1182. doi: 10.22034/APJCP.2017.18.5.1177 PMID: 28610399 PMC5555520

[ref52] Shen J. H. Rudzicz F. “Detecting anxiety through Reddit” in *Proceedings of the Fourth Workshop on Computational Linguistics and Clinical Psychology—From Linguistic Signal to Clinical Reality*. (Association for Computational Linguistics), 58–65 (2017).

[ref53] Sisk B. A. Fasciano K. Block S. D. Mack J. W. (2020). Impact of cancer on school, work, and financial independence among adolescents and young adults. Cancer 126, 4400–4406. doi: 10.1002/cncr.33081, PMID: 32658324 PMC7719071

[ref54] Smith H. R. (2015). Depression in cancer patients: pathogenesis, implications and treatment (review). Oncol. Lett. 9, 1509–1514. doi: 10.3892/ol.2015.2944, PMID: 25788991 PMC4356432

[ref55] Soiferman L. K. Compare and contrast inductive and deductive research approaches. (2010).

[ref56] Soleimani M. A. Bahrami N. Allen K.-A. Alimoradi Z. (2020). Death anxiety in patients with cancer: a systematic review and meta-analysis. Eur. J. Oncol. Nurs. 48:101803. doi: 10.1016/j.ejon.2020.101803, PMID: 32836000

[ref57] Stark D. P. House A. (2000). Anxiety in cancer patients. Br. J. Cancer 83, 1261–1267. doi: 10.1054/bjoc.2000.1405, PMID: 11044347 PMC2408796

[ref58] Syrowatka A. Motulsky A. Kurteva S. Hanley J. A. Dixon W. G. Meguerditchian A. N.. (2017). Predictors of distress in female breast cancer survivors: a systematic review. Breast Cancer Res. Treat. 165, 229–245. doi: 10.1007/s10549-017-4290-9, PMID: 28553684 PMC5543195

[ref59] Thomas D. (2006). A general inductive approach for analyzing qualitative evaluation data. Am. J. Eval. 27, 237–246. doi: 10.1177/1098214005283748

[ref60] Tripathi S. D. Parker P. D. Prabhu A. V. Thomas K. Rodriguez A. (2022). An examination of patients and caregivers on Reddit navigating brain Cancer: content analysis of the brain tumor Subreddit. JMIR Cancer 8:e35324. doi: 10.2196/35324, PMID: 35731559 PMC9260533

[ref61] Wang S.-Y. Hsu S. H. Gross C. P. Sanft T. Davidoff A. J. Ma X.. (2016). Association between time since Cancer diagnosis and health-related quality of life: a population-level analysis. Value Health 19, 631–638. doi: 10.1016/j.jval.2016.02.010, PMID: 27565280 PMC5002308

[ref62] Welcome to LIWC-22 (2022). Available at: https://www.liwc.app/

[ref63] Williams A. M. Khan C. P. Heckler C. E. Barton D. L. Ontko M. Geer J.. (2021). Fatigue, anxiety, and quality of life in breast cancer patients compared to non-cancer controls: a nationwide longitudinal analysis. Breast Cancer Res. Treat. 187, 275–285. doi: 10.1007/s10549-020-06067-6, PMID: 33392843 PMC8080260

[ref64] Yi J. C. Syrjala K. L. (2017). Anxiety and depression in Cancer survivors. Med. Clin. North Am. 101, 1099–1113. doi: 10.1016/j.mcna.2017.06.005, PMID: 28992857 PMC5915316

[ref65] Zubin J. Spring B. (1977). Vulnerability: a new view of schizophrenia. J. Abnorm. Psychol. 86, 103–126. doi: 10.1037/0021-843X.86.2.103 858828

[ref66] Zuckerman M. (1999). “Diathesis-stress models” in Vulnerability to Psychopathology: A Biosocial Model (American Psychological Association), 3–23. doi: 10.1037/10316-001

