# Supplementary material for: Mental health at different stages of cancer survival: a natural language processing study of Reddit posts
Source: Front Psychol. 2023 Jun 23;14:1150227. doi: 10.3389/fpsyg.2023.1150227 (PMC10326387; doi:10.3389/fpsyg.2023.1150227)
Supplement: Supplementary file 1 [file Data_Sheet_1.PDF]

1. The R code employed in running the global multivariate non-parametric analysis

```
library(npmv)

data_nlp = read.csv("Insert the path to the .csv file that serves as database")

nonpartest(dependent_variable_1|dependent_variable_2|...|
dependent_variable_X~independent_variable,data_nlp,permreps=1000)
```

2.The R code employed in running the chi^2 analysis and generating associated barplots

```
library(ggstatsplot)

data <- read.csv("Insert the path to the .csv file that serves as database")

ggbarstats(
  data,
  x = dominant_topic,
  y = group,
  bf.message = FALSE,
  label = "both"
)
```

3. Scrape cancer words

```
import requests
from bs4 import BeautifulSoup
from requests_html import HTMLSession
import unidecode
import json
import requests
import time
from selenium.webdriver.chrome.options import Options
import urllib
from selenium.webdriver.common.by import By
from string import ascii_lowercase
import re

url1 = 'https://www.cancer.org/cancer/glossary.html'

from selenium import webdriver
from selenium.webdriver.common.keys import Keys

options = Options()
options.add_argument('--headless')
options.add_argument('--disable-gpu')

driver = webdriver.Chrome(options = options)
driver.get(url1)
```

```
html = driver.execute_script("return document.getElementsByTagName('html')[0].innerHTML")

soup = BeautifulSoup(html, 'html.parser')
table = soup.find_all("div", {"class": "term"})

all_terms_url1 = []
for term in table:
    all_terms_url1.append(term.find("h3").getText().split('\n')[0])

all_terms_url1 = [re.sub(r'\[(.*?)\]', '', t) for t in all_terms_url1]
driver.close()

all_terms_url1 = [t.lower() for t in all_terms_url1]
print('before duplicates removal', len(all_terms_url1))
all_terms_url1 = list(set(all_terms_url1))
all_terms_url1 = sorted(all_terms_url1)

print('after duplicates removal', len(all_terms_url1))

with open('data/cancer.txt', 'wt') as f:
    for item in all_terms_url1:
        f.write(f'{item}\n')
```

4. Get the frequency of cancer words

```
import numpy as np
import pandas as pd
import tqdm
import regex
import re
import string
import argparse

parser = argparse.ArgumentParser(description='args')
parser.add_argument('--split', type = str, required = True)
parser.add_argument('--group', type = str, required = True)

args = parser.parse_args()
split = args.split
group = args.group

def get_cancer_words(cancer_terms, text):

    text = text.translate(str.maketrans('', '', string.punctuation))
    cancer_terms = [term.translate(str.maketrans('', '', string.punctuation)) for term in cancer_terms if
term != '']
    cancer_terms = '|'.join(["\\b" + t.strip() + "\\b" for t in cancer_terms])
    cancer_count = 0

    result_depr = re.findall(rf'{cancer_terms}', text)
    if len(result_depr):
```

```
        cancer_count += len(result_depr)

    return cancer_count
```

# Extract the frequencies for LIWC features

```
import numpy as np
import pandas as pd
import tqdm
import regex
import re
import string
import argparse

parser = argparse.ArgumentParser(description='args')
parser.add_argument('--split', type = str, required = True)
parser.add_argument('--group', type = str, required = True)

args = parser.parse_args()
split = args.split
group = args.group

def get_liwc(liwc_terms, text):

    liwc_terms = [term for term in liwc_terms if term != '']
    liwc_terms = '|'.join(["\\b" + t.strip() + "\\b" for t in liwc_terms])
    liwc_terms = liwc_terms.replace('*\\b', '')
    liwc_count = 0
    result_depr = re.findall(rf'{liwc_terms}', text.lower())
    if len(result_depr):
        liwc_count += len(result_depr)

    return liwc_count


df = pd.read_csv(f'data/survey/{split}/{group}.csv')
df = df.replace(np.nan, '', regex=True)

for i, row in tqdm.tqdm(df.iterrows(), total = len(df.index)):
    for liwc_feat in ['NEGEMO', 'POSEMO', 'ACHIEVE', 'BODY', 'DEATH', 'EATING', 'GROOM',
'HOME', 'JOB', 'MONEY', 'MUSIC',  'RELIG', 'SCHOOL', 'SEXUAL', 'SLEEP', 'SPORTS', 'TV', 'ANX',
'OPTIM']:

        with open(f'./data/LIWC/{liwc_feat}.txt', 'r') as f:
            liwc_terms = f.read().split('\n')
        text = row['text'] + ' ' + row['title']

        text = text.lower()
        length = row['word_count']

        liwc_count = get_liwc(liwc_terms, text)
        df.loc[i, f'{liwc_feat}_count'] = liwc_count
```

```
        df.loc[i, f'{liwc_feat}_freq'] = round(liwc_count/length, 4) * 100 if length else 0

df = df[df.columns.drop(list(df.filter(regex='freq')))]
df = df.drop(columns = ['text', 'title'])
df.to_csv(f'data/survey/{split}/{group}.csv', index = False)
```

5. Train model for depression detection

```
from transformers import BertForSequenceClassification, AdamW
import pandas as pd
import numpy as np
import argparse
from torch.utils.data import DataLoader, RandomSampler, SequentialSampler
import os
import random
import tqdm
from transformers import BertTokenizerFast
import torch
from torch.utils.data import TensorDataset, random_split
from sklearn.metrics import precision_score, recall_score, f1_score
from sklearn.model_selection import train_test_split
from transformers import get_linear_schedule_with_warmup
from sklearn.model_selection import train_test_split, StratifiedKFold, GridSearchCV
from sklearn import metrics
import glob

OUTPUT_DIR = './models/'
N_CLASSES = 2
EPOCHS = 1
BATCH_SIZE = 32

device = torch.device('cuda')

tokenizer = BertTokenizerFast.from_pretrained('bert-base-uncased', do_lower_case = True)

def get_train_data():
    blogs_depression = glob.glob('../data/Data_Collector/blogs_depression/*.txt')
    mixed_depression = glob.glob('../data/Data_Collector/mixed_depression/*.txt')
    reddit_depression = glob.glob('../data/Data_Collector/reddit_depression/*.txt')
    blogs_non_depression = glob.glob('../data/Data_Collector/blogs_non_depression/*.txt')
    mixed_non_depression = glob.glob('../data/Data_Collector/mixed_non_depression/*.txt')
    reddit_non_depression = glob.glob('../data/Data_Collector/reddit_non_depression/*.txt')
    reddit_breastcancer = glob.glob('../data/Data_Collector/reddit_breastcancer/*.txt')

    depression_files = blogs_depression + mixed_depression + reddit_depression
    non_depression_files = blogs_non_depression + mixed_non_depression + reddit_non_depression
+ reddit_breastcancer

    depression_texts = []
    for file in depression_files:
        with open(file, 'r', encoding="utf8", errors='ignore') as f:
            text = f.read()
```

```
        depression_texts.append(text)
    depression_labels = [1] * len(depression_texts)

    non_depression_texts = []
    for file in non_depression_files:
        with open(file, 'r', encoding="utf8", errors='ignore') as f:
            text = f.read()
        non_depression_texts.append(text)
    non_depression_labels = [0] * len(non_depression_texts)

    X = depression_texts + non_depression_texts
    y = depression_labels + non_depression_labels

    return np.array(X), np.array(y)

def preprocess(X, y):
    attention_masks = []
    input_ids = []

    for text in tqdm.tqdm(X, total = len(X)):
        encoded_dict = tokenizer.encode_plus(
            text,
            add_special_tokens = True,
            max_length = 256,
            padding = 'max_length',
            truncation = True,
            return_attention_mask = True,
            return_tensors = 'pt',
        )
        input_ids.append(encoded_dict['input_ids'])
        attention_masks.append(encoded_dict['attention_mask'])

    input_ids = torch.cat(input_ids, dim = 0)
    attention_masks = torch.cat(attention_masks, dim = 0)
    labels = torch.tensor(y)

    return input_ids, attention_masks, labels

def save_model(model, epoch, val_loss, val_f1):
    if not os.path.exists(OUTPUT_DIR):
        os.makedirs(OUTPUT_DIR, exist_ok = True)

    name = f"bert-epoch={epoch}-val_loss={np.round(val_loss, 3)}-f1={np.round(val_f1, 3)}"
    print(f"Saving model to {OUTPUT_DIR}/{name}" )
    model_to_save = model.module if hasattr(model, 'module') else model
    torch.save(model.state_dict(), f"{OUTPUT_DIR}/{name}")


def k_fold_cross_validation(X, y):

    skf = StratifiedKFold(n_splits = 5, random_state = 42, shuffle = True)
    fold = 0
```

```
train_scores = []
val_scores = []
precision_scores = []
recall_scores = []
f1_scores = []
for train_index, test_index in skf.split(X, y):
   fold += 1

   X_train, X_test = X[train_index], X[test_index]
   y_train, y_test = y[train_index], y[test_index]

   train_dataset = TensorDataset(*preprocess(X_train, y_train))
   test_dataset = TensorDataset(*preprocess(X_test, y_test))

   model = BertForSequenceClassification.from_pretrained(
      'bert-base-uncased',
      num_labels = N_CLASSES,
      output_attentions = False,
      output_hidden_states = False
   )

   model = torch.nn.DataParallel(model)
   model.to(device)

   train_dataloader = DataLoader(
      train_dataset,
      batch_size = BATCH_SIZE,
      shuffle = True
   )

   test_dataloader = DataLoader(
      test_dataset,
      batch_size = BATCH_SIZE,
      shuffle = False
   )

   optimizer = AdamW(
      model.parameters(),
      lr = 2e-5,
   )

   total_steps = len(train_dataloader) * EPOCHS

   scheduler = get_linear_schedule_with_warmup(optimizer, num_warmup_steps = 0,
num_training_steps = total_steps)

   for epoch in range(EPOCHS):
      total_train_loss = 0
      model.train()

      pbar = tqdm.tqdm(train_dataloader)
```

```
for step, batch in enumerate(pbar):

    b_input_ids = batch[0].to(device)
    b_input_mask = batch[1].to(device)
    print('batch', batch[2])
    b_labels = batch[2].view((-1, 1)).to(device)
    print('batch labels', b_labels)

    optimizer.zero_grad()

    output = model(b_input_ids,
        token_type_ids=None,
        attention_mask=b_input_mask,
        labels=b_labels
    )
    loss = output.loss.mean()
    print(output.logits)

    total_train_loss += loss.item()
    loss.backward()

    torch.nn.utils.clip_grad_norm_(model.parameters(), 1.0)

    optimizer.step()
    scheduler.step()

    y_true = b_labels.detach().cpu().numpy().ravel()
    y_pred = output.logits.detach().argmax(axis = -1).cpu().numpy().ravel()

    acc = (y_true == y_pred).mean()
    f1 = f1_score(y_true, y_pred)
    pbar.set_description(f'Epoch={epoch} | batch_acc={acc} | batch_f1={np.round(f1, 3)}')

avg_train_loss = total_train_loss / len(train_dataloader)

model.eval()

total_eval_accuracy = 0
total_eval_loss = 0
total_eval_f1 = 0

print("Running validation")
y_pred = []
y_true = []
for batch in tqdm.tqdm(test_dataloader, total = len(test_dataloader)):
    b_input_ids = batch[0].to(device)
    b_input_mask = batch[1].to(device)
    b_labels = batch[2].to(device)

    with torch.no_grad():
      output = model(b_input_ids,
      token_type_ids=None,
```

```
            attention_mask=b_input_mask,
            labels=b_labels)

          total_eval_loss += output.loss.mean().item()

          logits = output.logits.detach().argmax(dim = -1).cpu().numpy().ravel()

          label_ids = b_labels.cpu().numpy().ravel()
          y_pred.extend(logits.tolist())
          y_true.extend(label_ids.tolist())

          total_eval_accuracy += (logits == label_ids).mean()

        avg_val_accuracy = total_eval_accuracy / len(test_dataloader)
        avg_val_loss = total_eval_loss / len(test_dataloader)

        f1_epoch = f1_score(y_true, y_pred)
        precision_epoch = precision_score(y_true, y_pred)
        recall_epoch = recall_score(y_true, y_pred)

        print("  Accuracy: {0:.4f}".format(avg_val_accuracy), "F1: {0:.4f}".format(f1_epoch), "Precision:
{0:.4f}".format(precision_epoch), "Recall: {0:.4f}".format(recall_epoch))
        save_model(model = model, epoch = epoch, val_loss = avg_val_loss, val_f1 = f1_epoch)

      val_scores.append(avg_val_accuracy)
      precision_scores.append(precision_epoch)
      recall_scores.append(recall_epoch)
      f1_scores.append(f1_epoch)

    print('Average results')
    print("Accuracy", np.mean(val_scores))
    print("Precision", np.mean(precision_scores))
    print("Recall", np.mean(recall_scores))
    print("F1 score", np.mean(f1_scores))

X, y = get_train_data()

k_fold_cross_validation(X, y)
```

6. Code for prediction using the depression model

```
import numpy as np
import pandas as pd
import argparse
import tqdm
import emoji
import regex
from textblob import TextBlob
from transformers import BertForSequenceClassification, AdamW
from torch.utils.data import DataLoader
import os
```

```
import random
from transformers import BertTokenizerFast
import torch
from torch.utils.data import TensorDataset, random_split
from sklearn.metrics import precision_score, recall_score, f1_score
from sklearn.model_selection import train_test_split
import torch.nn.functional as F
import re
import spacy

parser = argparse.ArgumentParser(description='Do stuff.')
parser.add_argument('--split', type = str, required = True)
parser.add_argument('--group', type = str, required = True)
parser.add_argument('--depression_flag', type = bool, required = False)

args = parser.parse_args()
split = args.split
group = args.group
depression_flag = args.depression_flag

nlp = spacy.load("en_core_web_sm", disable = ['ner', 'parser'])

device = torch.device('cuda')
N_CLASSES = 2
BATCH_SIZE = 512

def get_terms(text, kind):
    with open(f'./data/{kind}.txt', 'r') as f:
        terms = f.read().split('\n')
    terms = [term for term in terms if term != '']
    count_terms = 0
    for term in terms:
        result = re.findall(rf'\b{term}', text)
        if len(result):
            count_terms += len(result)
    return count_terms

tokenizer = BertTokenizerFast.from_pretrained('bert-base-uncased', do_lower_case = True)

model = BertForSequenceClassification.from_pretrained(
    'bert-base-uncased',
    num_labels = N_CLASSES,
    output_attentions = False,
    output_hidden_states = False
)

def preprocess(df):
    attention_masks = []
    input_ids = []
    idx = []

    for i, row in tqdm.tqdm(df.iterrows(), total = len(df.index)):
```

```
        encoded_dict = tokenizer.encode_plus(
            row['text'],
            add_special_tokens = True,
            max_length = 256,
            pad_to_max_length = True,
            return_attention_mask = True,
            return_tensors = 'pt',
        )
        input_ids.append(encoded_dict['input_ids'])
        attention_masks.append(encoded_dict['attention_mask'])
        idx.append(torch.tensor([i]))

    input_ids = torch.cat(input_ids, dim = 0)
    attention_masks = torch.cat(attention_masks, dim = 0)
    idx = torch.cat(idx, dim = 0)

    return input_ids, attention_masks, idx

def get_depression(split, group, df, model):

    df['text_full'] = df[['text', 'title']].apply(lambda x: ' '.join(x.dropna()), axis=1)
    preds_dataset = TensorDataset(*preprocess(df))

    state_dict = torch.load('depression/models/bert-epoch=0-val_loss=0.245-f1=0.912')

    state_dict = {k.replace('module.', ''): v for k,v in state_dict.items()}
    model.load_state_dict(state_dict)
    model = torch.nn.DataParallel(model)
    model.to(device)
    model.eval()

    preds_dataloader = DataLoader(
        preds_dataset,
        batch_size = BATCH_SIZE,
        shuffle = False
    )

    with torch.no_grad():
        out = []
        for step, batch in enumerate(tqdm.tqdm(preds_dataloader)):
            b_input_ids = batch[0].to(device)
            b_input_mask = batch[1].to(device)

            output = model(b_input_ids,
                token_type_ids=None,
                attention_mask=b_input_mask)

            output = F.softmax(output.logits, dim = 1).cpu().numpy()[:, 1].tolist()
            out.extend(output)

    df['depression_score'] = out
    out_flag = [1 if o >= 0.5 else 0 for o in out]
```

```
    df['depression_flag'] = out_flag

    df.to_csv(f'data/survey/{split}/{group}_depression.csv', index = False)

if depression_flag:
    df = pd.read_csv(f'data/survey/{split}/{group}.csv')
    df = df.replace(np.nan, '', regex=True)
    get_depression(split, group, df, model)
else:
    df = pd.read_csv(f'data/survey/{split}/{group}.csv')
    df = df.replace(np.nan, '', regex=True)
    get_sentiment_user_level(split, group, df)
```

7. Train LDA model

```
import numpy as np
import pandas as pd
from gensim.models import LdaModel, LdaMulticore
from gensim.test.utils import common_dictionary, common_corpus
from gensim.corpora import Dictionary
from gensim.matutils import corpus2dense
from gensim.models.coherencemodel import CoherenceModel
from gensim.models import Phrases
import matplotlib.pyplot as plt
import pickle
import preprocessor as p
import spacy
import re
from nltk.corpus import stopwords
from gensim.utils import simple_preprocess
from collections import Counter

import logging
logging.basicConfig(format='%(asctime)s : %(levelname)s : %(message)s', level=logging.INFO)

nlp = spacy.load("en_core_web_sm", disable = ['ner', 'parser'])

stop_words = stopwords.words('english')
with open('stopwords.txt', 'rt') as f:
    words = [w.strip() for w in f.readlines()]
stop_words.extend(words)

def get_lemmas(text):
    lemmas = [token.lemma_ for token in nlp(text) if token.pos_ == 'NOUN']
    return ' '.join(lemmas)

def preprocess_data(df):
    print('Preprocessing data...')
    letter_regex = re.compile(r'(@[A-Za-z_]+)|([^A-Za-z ]+)')
    url_regex = re.compile(r'http[s]?://\S+')
    # text = df['text_nouns']
    text = df['text_full']
```

```
    text = [get_lemmas(t) for t in text]
    text = [url_regex.sub(' <url> ', t) for t in text]
    text = [url_regex.sub('', t) for t in text]
    text = [p.clean(letter_regex.sub(' ', t)).lower() for t in text]

    return text

def remove_stopwords(texts):
    return [[word for word in simple_preprocess(str(doc)) if word not in stop_words] for doc in texts]

def lda(mental_health_class):
    short_term = pd.read_csv('data/survey/short_term.csv')
    transition = pd.read_csv('data/survey/transition.csv')
    long_term = pd.read_csv('data/survey/long_term.csv')

    if mental_health_class == 'depression':
        short_term = short_term[short_term['depression_flag'] == 1]
        transition = transition[transition['depression_flag'] == 1]
        long_term = long_term[long_term['depression_flag'] == 1]
    elif mental_health_class == 'anxiety':
        short_term = short_term[short_term['ANX'] > 0]
        transition = transition[transition['ANX'] > 0]
        long_term = long_term[long_term['ANX'] > 0]

    data_df = pd.DataFrame(np.concatenate([short_term, transition, long_term]),
columns=before_diagnosis.columns)

    data_df = data_df[data_df['text_nouns'].notna()]

    text = preprocess_data(data_df)
    tokenized = remove_stopwords(text)

    bigram_phraser = Phrases(tokenized)
    tokenized = [bigram_phraser[t] for t in tokenized]
    with open(f'checkpoints/models_lda/lda_{mental_health_class}/bigram_phraser.pkl', 'wb') as f:
        pickle.dump(bigram_phraser, f)

    trigram_phraser = Phrases(bigram_phraser[tokenized])
    tokenized = [trigram_phraser[t] for t in tokenized]
    with open(f'checkpoints/models_lda/lda_{mental_health_class}/trigram_phraser.pkl', 'wb') as f:
        pickle.dump(trigram_phraser, f)

    with open(f'checkpoints/models_lda/lda_{mental_health_class}/tokenized_text.pkl', 'wb') as f:
        pickle.dump(tokenized, f)

    dictionary = Dictionary(tokenized)
    dictionary.filter_extremes(no_below=10, no_above=0.1)
    corpus = [dictionary.doc2bow(line) for line in tokenized]

    with open('checkpoints/models_lda/lda_{mental_health_class}/dictionary.pkl', 'wb') as f:
        pickle.dump(dictionary, f)
    with open('checkpoints/models_lda/lda_{mental_health_class}/corpus.pkl', 'wb') as f:
```

```
        pickle.dump(corpus, f)

    def compute_coherence_values(dictionary, corpus, texts, limit, start, step):
        coherence_values = []
        model_list = []
        for num_topics in range(start, limit, step):
            print(num_topics)
            model=LdaMulticore(corpus=corpus, id2word=dictionary, random_state=100,
num_topics=num_topics, chunksize = 5000, passes = 10,  workers=15)
            model_list.append(model)
            coherencemodel = CoherenceModel(model=model, texts=texts, dictionary=dictionary,
coherence='c_v')
            coherence_values.append(coherencemodel.get_coherence())
        return model_list, coherence_values

    start = 5
    limit = 40
    step = 5

    model_list, coherence_values = compute_coherence_values(dictionary=dictionary, corpus=corpus,
texts=tokenized, start=start, limit=limit, step=step)

    for idx in range(len(model_list)):
        with
open(f'checkpoints/models_lda/lda_{mental_health_class}/lda_{model_list[idx].num_topics}_{coheren
ce_values[idx]}.pkl', 'wb') as f:
            pickle.dump(model_list[idx], f)
        print(model_list[idx], coherence_values[idx])

    x = range(start, limit, step)
    plt.plot(x, coherence_values)
    plt.xlabel("Num Topics")
    plt.ylabel("Coherence score")
    plt.legend(("coherence_values"), loc='best')
    plt.savefig(F'topics-5_40_{mental_health_class}.png')
    plt.show()

lda(mental_health_class = 'depression')
lda(mental_health_class = 'anxiety')
```

8. Extract topics for each post

```
import numpy as np
import pandas as pd
from gensim.models import LdaModel, LdaMulticore
from gensim.test.utils import common_dictionary, common_corpus
from gensim.corpora import Dictionary
from gensim.matutils import corpus2dense
from gensim.models.coherencemodel import CoherenceModel
from gensim.models import Phrases
import matplotlib.pyplot as plt
import pickle
```

```
import preprocessor as p
import re
from nltk.corpus import stopwords
from gensim.utils import simple_preprocess
from collections import Counter
import spacy

nlp = spacy.load("en_core_web_sm", disable = ['ner', 'parser'])

stop_words = stopwords.words('english')
with open('stopwords.txt', 'rt') as f:
	words = [w.strip() for w in f.readlines()]
stop_words.extend(words)

def get_lemmas(text):
	lemmas = [token.lemma_ for token in nlp(text) if token.pos_ == 'NOUN']
	return ' '.join(lemmas)

def preprocess_data(df):
	print('Preprocessing data...')
	letter_regex = re.compile(r'(@[A-Za-z_]+)|([^A-Za-z ]+)')
	url_regex = re.compile(r'http[s]?://\S+')
	text = df['text_full']
	text = [get_lemmas(t) for t in text]
	text = [url_regex.sub(' <url> ', t) for t in text]
	text = [url_regex.sub('', t) for t in text]
	text = [p.clean(letter_regex.sub(' ', t)).lower() for t in text]

	return text

def remove_stopwords(texts):
	return [[word for word in simple_preprocess(str(doc)) if word not in stop_words] for doc in
texts]

def format_topics_sentences(ldamodel, corpus, df):
	sent_topics_df = pd.DataFrame()

	# Get main topic in each document
	for i, row in enumerate(corpus):
		topics = ldamodel[row]
		topics = sorted(topics, key=lambda x: (x[1]), reverse=True)

		# Get the Dominant topic, Perc Contribution and Keywords for each document
		for j, (topic_num, prop_topic) in enumerate(topics):
			if j == 0:  # => dominant topic
				wp = ldamodel.show_topic(topic_num)
				topic_keywords = ", ".join([word for word, prop in wp])
				sent_topics_df = sent_topics_df.append(pd.Series([int(topic_num),
round(prop_topic,4), topic_keywords]), ignore_index=True)
			else:
				break
	sent_topics_df.columns = ['dominant_topic', 'perc_contribution', 'topic_Keywords']
```

```
        # Add original text
        full_text = pd.Series(df['text_full'].values)
        username = pd.Series(df['username'].values)
        post_id = pd.Series(df['post_id'].values)
        date = pd.Series(df['date'].values)

        sent_topics_df = pd.concat([sent_topics_df, full_text, username, post_id, date], axis=1)
        return(sent_topics_df)


def get_topics():
        short_term_path = 'data/survey/short_term_depression.csv'
        transition_path = 'data/survey/transition_depression.csv'
        long_term_path = 'data/survey/long_term_depression.csv'

        with open(f'checkpoints/models_lda/lda_depression/bigram_phraser.pkl', 'rb') as f:
                bigram_phraser = pickle.load(f)

        with open(f'checkpoints/models_lda/lda_depression/trigram_phraser.pkl', 'rb') as f:
                trigram_phraser = pickle.load(f)

        with open('checkpoints/models_lda/lda_depression/dictionary.pkl', 'rb') as f:
                dictionary = pickle.load(f)

        with open(f'checkpoints/models_lda/lda_depression/lda.pkl', 'rb') as f:
                lda_model = pickle.load(f)

        # for idx, topic in lda_model.show_topics(formatted=False, num_words= 10):
        #         print('Topic: {} \nWords: {}'.format(idx, [w[0] for w in topic]))
        # print(lda_model.print_topics())

        for df_path in [short_term_path, transition_path, long_term_path]:
                df = pd.read_csv(df_path)
                df = df[df['depression_flag'] == 1]

                df = df.replace(np.nan, '', regex=True)
                texts = df['text_full'].tolist()
                tokenized = preprocess_data(df)
                tokenized = remove_stopwords(tokenized)
                tokenized = [bigram_phraser[t] for t in tokenized]
                tokenized = [trigram_phraser[t] for t in tokenized]
                corpus = [dictionary.doc2bow(line) for line in tokenized]

                df_topic_sents_keywords = format_topics_sentences(ldamodel=lda_model,
corpus=corpus, df = df)
                df_dominant_topic = df_topic_sents_keywords.reset_index()
                df_dominant_topic.columns = ['index', 'dominant_topic', 'topic_perc_contrib',
'keywords', 'text_full', 'username', 'post_id', 'date']
                df_dominant_topic.to_csv(f'data/survey/topics/{df["group"].unique()[0]}.csv', index =
False)
```

```
def get_most_representative_texts():
	groups = ['short_term', 'transition', 'long_term']
	df_final = []
	for group in groups:
		df = pd.read_csv(f'data/survey/topics/{group}.csv')
		df_final.append(df)

	df_final = pd.concat(df_final)
	df_final.sort_values(['topic_perc_contrib', 'dominant_topic'], ascending=[True, True])
	df_final.to_csv('data/survey/topics/concatenated.csv')


def get_topic_percentage():
	groups = ['short_term', 'transition', 'long_term']
	for group in groups:
		df = pd.read_csv(f'data/survey/{group}.csv')
		aggregate = df.groupby('dominant_topic').agg({'index': 'count'}).reset_index()
		aggregate['perc'] = 100 * aggregate['index'] / aggregate['index'].sum()

get_topic_percentage()
```
